# Effects of couples positive massage programme on wellbeing, perceived stress and coping, and relation satisfaction*

**DOI:** 10.1080/21642850.2019.1682586

**Published:** 2019-10-23

**Authors:** Sayuri M. Naruse, Mark Moss

**Affiliations:** Department of Psychology, Northumbria University, Newcastle upon Tyne, UK

**Keywords:** Wellbeing, stress, coping, couples massage, self-care

## Abstract

**Objectives:** Although supporting preventative self-regulation and self-care activity for daily stress is important as dyadic coping, there seems a paucity of exploration of non-verbal interventions such as tactile communication. This preliminary experimental study assessed the efficacy of a short educational massage programme for healthy but stressed couples. The study aimed to investigate if the educational mutual massage (Positive Massage) programme has any acute and sustained effects on wellbeing, perceived stress and coping, and relationship satisfaction among couples.

**Design:** A pseudo randomised two group design employing a delayed treatment element assessed the effects of the Positive Massage programme and subsequent at-home application. Thirty-eight participants completed a three-week massage course.

**Main Outcome Measures:** Measurements of the Warwick-Edinburgh Mental Well-being Scale, the Rhode Island Stress and Coping Inventory, and the Positive Feelings Questionnaire were collected online using Qualtrics at three time points (the start, the end, and three weeks after the course). Data were analysed with mixed ANOVAs.

**Results:** Mental wellbeing, and perceived stress and coping significantly improved from before to after the Positive Massage programme. There was no significant decline after the cessation of the massage programme. Relationship satisfaction did not show significant changes from the initial assessment.

**Conclusions:** The overall effects of the Positive Massage programme indicate the importance of developing further large scale studies of mutual massage as a safe and beneficial self-care activity. This innovative study has laid the groundwork for future studies into the possibility of mutual massage as a self-regulation dyadic coping strategy for home use to improve overall wellbeing.

## Introduction

Decades of research has revealed that stress, especially chronic daily hassles stress, can be detrimental to health and wellbeing (DeLongis, Folkman, & Lazarus, 1988; Serido, Almeida, & Wethington, 2004). Globally, there is also an increased emphasis on the prevention of ill health by reducing stress, and through empowering self-regulation and supporting self-care, given that stress/lifestyle related diseases are highly prevalent and burdensome but also preventable (World Health Organization Regional Office for Europe, 2013).

Stress is the discrepancy between a person’s resources and environmental demands, and the impact of stress depends on the appraisal of perceived stress and coping ability (Lazarus & Folkman, 1984). In addition to one’s own perceived stress and coping ability, the experiences of an individual’s partner can also affect psychological and physical wellbeing and health (Buck & Neff, 2012; Chopik & O’Brien, 2017; Falconier, Nussbeck, Bodenmann, Schneider, & Bradbury, 2015). Furthermore, stress levels can negatively influence couples interactions in daily life (Doerr, Nater, Ehlert, & Ditzen, 2018), and the quality of the relationship – as measured by communication problems and relationship satisfaction (Buck & Neff, 2012; Falconier et al., 2015). Therefore, interpersonal factors, as well as individual coping systems, should be considered as targets for effective interventions to combat the impact of stress (Samios & Baran, 2018). A range of dyadic coping strategies^1^ have been identified (Falconier et al., 2015), including understanding the other person’s perspective regarding stress, and couple’s communication, coordination and collaboration regarding daily management tasks e.g. couples coping enhancement training (Bodenmann & Shantinath, 2004). However, there seems a paucity of exploration regarding non-verbal interventions such as tactile communication.

Interestingly, stress-buffering effects of touch have previously been demonstrated among couples (Ditzen et al., 2007; Jakubiak & Feeney, 2017) during periods of learning (Holt-Lunstad, Birmingham, & Light, 2008), conflict (Jakubiak & Feeney, 2019) and stressful times (Coan, Schaefer, & Davidson, 2006). It is proposed that touch provides psychological benefit between intimate partners by strengthening bonds that consequently enhance affect and wellbeing (Debrot, Schoebi, Perrez, & Horn, 2013). Moreover, a study investigating seven physical affection factors among romantic couples found that backrubs/massage was rated most favourably (Gulledge, Gulledge, & Stahmannn, 2003). Therefore, we propose a short (3 weeks, 1 h class per week) educational massage programme for couples as a putative coping intervention that might deliver mutual benefits. Developing such a simple preventative^2^ intervention that might increase each other’s wellbeing as well as strengthening coping mechanisms among stressed couples would be of considerable value. The importance of making such an intervention pleasant and easily achievable should not be underestimated when attempting to get ‘buy-in’ from those already experiencing high level of perceived stress in daily life.

Massage is one of the oldest forms of healthcare and has been reported to have beneficial effects on various physical and psychological conditions (Field, 2016; Moraska, Pollini, Boulanger, Brooks, & Teitlebaum, 2010), commonly used for cancer, palliative care and pain management (Alves, Gonçalves Jardim, & Pereira Gomes, 2017; Boitor, Gélinas, Richard-Lalonde, & Thombs, 2017; Crawford et al., 2016). Reported mental benefits of massage include improvements in distress level (Keir & Saling, 2012), decreased depression (Alves et al., 2017; Field, 2014), stress reduction (Turkeltaub, Yearwood, & Friedmann, 2014), anxiety reduction (Alves et al., 2017;Brand, Munroe, & Gavin, 2013), and the promotion of wellbeing (Alves et al., 2017; McFeeters, Pront, Cuthbertson, & King, 2016).

Importantly in the context of our current work, the great majority of massages in research have been applied by trained massage therapists or health professionals (e.g. physiotherapists) due to its use as a therapeutic intervention for a diagnosed problem. However, a relatively small but significant number of studies exist showing positive effects of massage by lay people such as partners (Field et al., 2008), significant others (Forchuk et al., 2004), carers (Collinge et al., 2013; Tuohy, Graham, Johnson, Tuohy, & Burke, 2015), and volunteers (Gensic, Smith, & LaBarbera, 2017). Interestingly, the effects of massage reported by lay caregivers (29–44% in symptom reduction) have been found to be very close to those from professional massage therapists (21–52%) (Collinge et al., 2013). Besides, and importantly, the safety of massage is deemed to be high, with reports of adverse consequences following massage largely limited to those resulting from the misuse of electrical massage devices (241 out of 256 cases) (Posadzki & Ernst, 2013). To the current knowledge of the authors the only paper dealing exclusively with the effects of mutual massage among couples derives from our current research (Naruse, Cornelissen, & Moss, 2018). This may be due to a lack of awareness, or the absence of an accessible massage style that might be easily incorporated into daily life among the general adult population in the UK. This is despite the fact that more than 200 styles of massage are practiced around the world.

The aims of the study were therefore to investigate:

1. If an educational massage programme (training at class + application at home) has any effects on mental wellbeing, perceived stress and coping, and relationship satisfaction among couples;
2. If there are any sustained effects after the completion of the massage programme at week 6; and
3. If healthy but stressed couples can learn and continue to apply massage with satisfaction at home in their daily lives.

To this end, a simple sequence of massage for home use, called Positive Massage (PM), was devised, and a short massage course (the PM programme) set up for people to learn and practice in close relationships (Naruse et al., 2018). Throughout the rest of this article, the term *massage* designates PM rather than other forms of massage.

Three distinctive elements in this preliminary experimental study were:

1. Hands of massage are moved from professional to lay people;
2. The setting of massage application is at home instead of public specialised facilities; and
3. A novel trial of two-way (exchange) short massage (15 min) among adult couples rather than one-way (just receiving), aiming for a benefit to both parties’ wellbeing rather than a therapeutic effect for the receivers.

## Methods

### Research design

**Figure 1.**
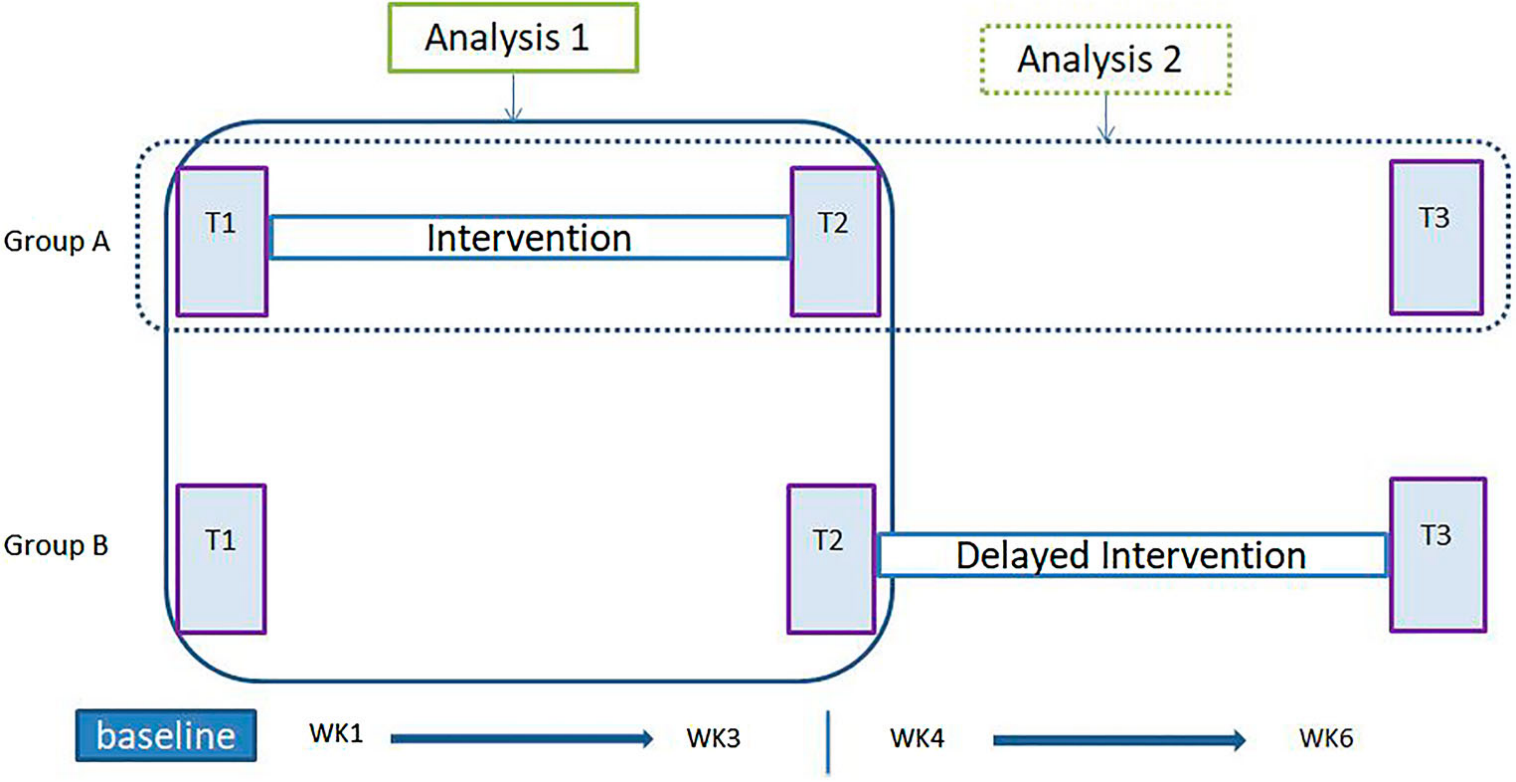
Time Flow Chart and Analysis.

This study took an experimental quantitative approach, employing two groups and comparing the effect of massage in a real-world setting through a delayed treatment design. The sample was allocated to either group A (intervention at week1–3) or B (Control group, no intervention week 1–3 but delayed intervention at week 4–6). There are both ‘between participants’ (groups A and B) and ‘within participants’ (repeated assessments) aspects of the data. The dataset was collected online using Qualtrics software (www.qualtrics.com) in the participants’ homes at the same three time-points (T1, T2 &T3) for both groups (Figure 1). SPSS version 24 (IBM, 2016) was used for data analysis. The study received ethical approval from The Faculty of Health and Life Sciences Research Ethics Committee at the University of Northumbria.

### Participants

**Figure 2.**
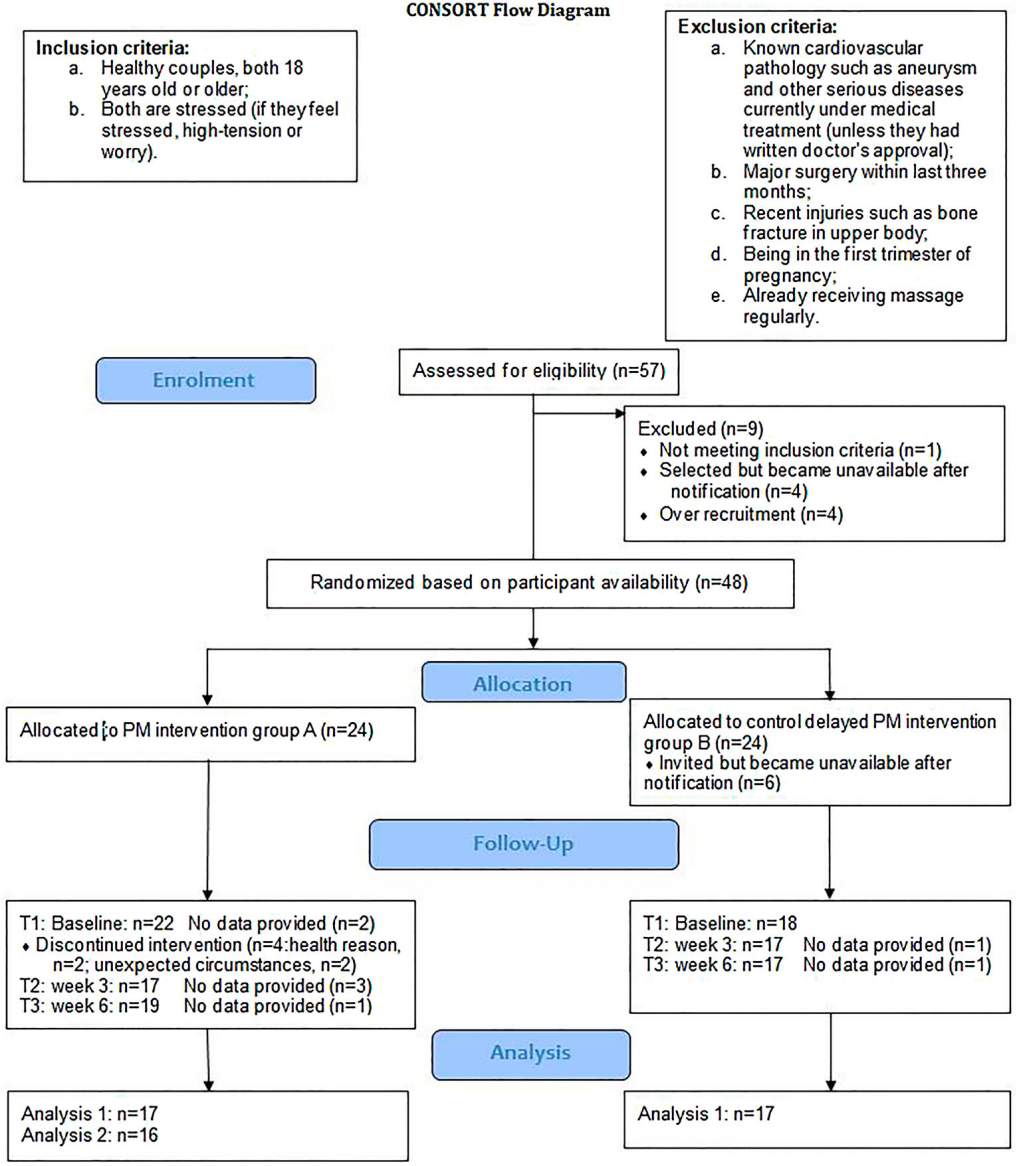
CONSORT Flow Chart.

Recruitment was by voluntary response sampling via posters, flyers, email and social media. The study’s inclusion and exclusion criteria are shown in Figure 1. As the Consolidated Standards of Reporting Diagram (CONSORT; see http://www.consort-statement.org/) (Figure 2) shows, 48 volunteers who agreed to take part were asked their availability/preference for the date of the PM course, and then randomly allocated 24 to group A (intervention) and 24 to group B (delayed intervention). Of these, 42 started the study and 38 continued through the three-week programme. Six participants in group B became unavailable during waiting for the delayed PM programme, and 4 participants in group A discontinued during the programme due to health reason (*n* = 2) and unexpected circumstances (*n* = 2). Not all the participants recorded the entire datasets at all time-points. Therefore, only 34 participants’ data were used for the analysis 1 and follow up frequency data 2&3, and 16 (i.e. group A) for analysis 2 (see Hypotheses and Figures 1 & 2). Participants’ mean age was 36.8 (SD = 10.3) and the mean length of relationship was 8.3 years (SD = 9.6). Fourteen couples were hetero-sexual and 3 couples were homo-sexual. As the comparative demographic data is depicted in Table 1, participants in group A and B did not differ. ANOVAs on the demographic variables show that the groups did not differ in terms of age *F* (1, 32) = .05, *p*= .83, or relationship length *F* (1, 32) = .15, *p *= .71.

**Table 1.** Demographic Data between Group A and B.

	Group A	Group B	Total
*Number of participants*			
Started	24	18	42
Completed (frequency data 1)	20	18	38
Data recorded (analysis 1&2, frequency data 2&3)	17	17	34
*Mean Age*	37.2 (SD 11.6)	36.4 (SD 9.1)	36.8 (SD 10.3)
*Gender*			
Male	7	8	15
Female	10	9	19
*Mean Time length of relationship/Year*	8.9 (SD 9.9)	7.6 (SD 9.5)	8.3 (SD 9.6)
*Marital status (% within group)*			
Married	9 (52.9%)	7 (41.2%)	16 (47.1%)
Cohabitant	2 (11.8%)	6 (35.3%)	8 (23.5%)
Other	6 (35.3%)	4 (23.5%)	10 (29.4%)
*Ethnicity (% within group)*			
White British	11 (64.7%)	10 (58.8%)	21 (61.8%)
White European	2 (11.8%)	4 (23.5%)	6 (17.6%)
Asian	1 (5.9%)	1 (5.9%)	2 (5.9%)
Other	1 (5.9%)	1 (5.9%)	2 (5.9%)
Prefer not to state	2 (11.8%)	1 (5.9%)	3 (8.8%)

### Instruments

The main data set included three questionnaires:

- The 14-item Warwick-Edinburgh Mental Well-being Scale (WEMWBS) (Tennant et al., 2007) to assess mental well-being;
- Ten items selected from the 12-item Rhode Island Stress and Coping Inventory (RISCI) (Fava, Ruggiero, & Grimley, 1998) to test perceived stress (Stress hereafter, 5-items) and perceived coping^3^ (Coping hereafter, 5-items). Two items (Item 5: pressurised by others; and item 6: stressed by unexpected events) were excluded because they are beyond potential massage effects. The 10 remaining items have been used in other stress-related studies (e.g. Evers et al., 2006; Horiuchi, Tsuda, & Kim, 2010).
- A modified 14-item version of the 17- item Positive Feelings Questionnaire (PFQ) (O’Leary, Fincham, & Turkewitz, 1983) was used to assess relationship satisfaction. Three items were excluded as these are not relevant to the effects of the current intervention because the items are related to perceptions of sexual relations and physical appearance.

The rationale behind the choice of these psychological tests was: brevity, simplicity, and quality of the contents. The WEMWBS has been used in a number of responsiveness validation studies (e.g. Bass, Dawkin, Muncer, Vigurs, & Bostock, 2016) and is frequently used across health science where high reliability has been reported for the UK population (*α* = .91). The RISCI is a well-used inventory in stress and health related studies especially in health behaviours (e.g. Kelly, Jonathon Rendina, Vuolo, Wells, & Parsons, 2015). Both subscales have shown adequate reliability, *α* = .82 in Stress and *α* = .81 in Coping. The PFQ has excellent reliability, *α* = .94, in measuring positive affections towards one’s spouse (O’Leary et al., 1983). A minor modification was applied to adapt the scale for all couples’ relationship status.

A massage log was also implemented online using Qualtrics in order to measure participants’ compliance to the massage regime at home during the PM programme. Questionnaires completed at the end of the PM programme included questions regarding intention to continue couples massage, feedback regarding exchanging massage, and whether PM would be recommended to others. Questionnaires for group A at T3 also included asking the frequency of massage during three weeks following completion of the PM programme.

### Intervention

The PM programme used in this study is a short 3 week programme that consists of a one-hour class each week and practice massage at home between classes. The programme was written originally in 2010 by the first author and developed into its current form for this study. The distinct characteristics of the PM programme are: 1) A short programme for those in close relationships to learn together; 2) Its aim to promote wellbeing by stress reduction and empower connection via skills of caring touch; 3) Simple massage easily applied at home without hassles (i.e. no need to remove clothing and no oil); and 4) First-hand learning and practice in the supervised classes to equip couples with the confidence and skills to practice/exchange massages at home. The philosophical base of the programme shares the principles of positive psychology (Seligman & Csikszentmihalyi, 2000), therefore the PM programme is proposed to be a positive activity intervention (Lyubomirsky & Layous, 2013). The programme contents cover the topics such as communication, sensitivity, safety, indications, and contraindications to massage, and awareness exercise – created to help the learner increase their sensitivity to their own body as well as the partners.

PM is a simple sequence of massage adapting uniquely fused styles from East and West. The focus of PM is not as a therapy for specific problems but rather on promoting wellbeing as a coping strategy with a preventative intention on a daily basis. The skills are relatively simple, but include important acupressure point and trigger point techniques for the effective application of massage. Participants are also guided to be sensitive to their partner’s body and feelings when giving the massage with the aid of verbal and non-verbal communications. For further details of PM intervention, please refer to Naruse et al. (2018).

### Procedure

Both group A and B participants were invited to the University of Northumbria to complete the PM programme. Each class was delivered by the first author helped by an assistant. The participants learned back massage (12 steps and 11 components) at the first class; arm, neck and head massage (11 steps and 9 new components) at the second class; and all parts plus face massage (17 steps and 5 new components) at the third class. A guide for a 15 min sequence for each practice was given to the participants with an allowance to adjust the duration, and being flexible as to the part of the body to apply massage to according to the partner’s need/preference. The participants were encouraged to carry out the massage practice ideally three times a week for three weeks at home. Previous studies of lay massage suggest three times a week massage at home is feasible (Collinge et al., 2013; Silva, Schalock, & Williams, 2013). To aid practice of the massage at home, handouts of the massage protocol were provided at each class.

### Hypotheses

Our hypotheses were:

1. There would be significant differences between group A and B at T2 on mental wellbeing, Stress and Coping, and relationship satisfaction (analysis 1);
2. There would be a sustained effect of the PM programme manifest at follow up (T3) in group A (analysis 2);
3. Participants who undertake the PM programme would have practiced PM (give a massage and receive a massage) an average of at least once per week at home during the programme (frequency data 1);
4. Participants who completed the PM programme in group A would go on to practice PM at least three times during three weeks after the programme finished (frequency data 2); and
5. Participants would indicate their intention to continue exchanging PM at T3 and also to recommend of PM to their friends and family (frequency data 3).

## Results

### Analysis 1 effect of intervention

**Figure 3.**
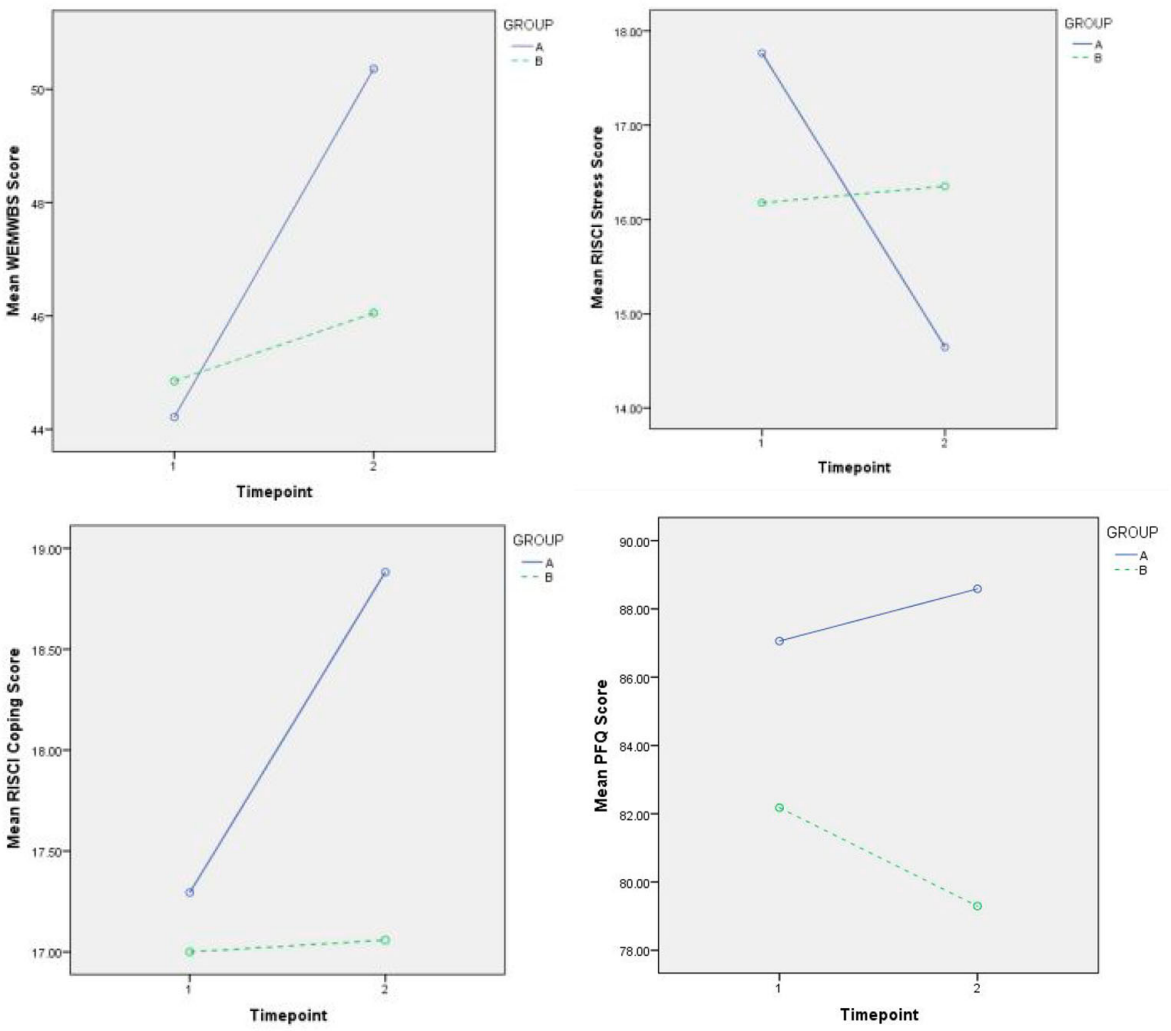
Interaction Effect between Group and Time-point on the WEMWBS Score, the RISCI Stress and Coping Score, and the PFQ Score (*N* = 34; group A, *n* = 17; group B, *n* = 17). WEMWBS = Warwick-Edinburgh Mental Well-being Scale (Between 14 and 70: 70 = most positive), RISCI Stress = Rhode Island Stress and Coping Inventory Stress subscale (Between 5 and 25: 25 = greatest perceived stress), RISCI Coping = RISCH Coping subscale (Between 5 and 25: 25 = greatest coping ability), PFQ = Positive Feelings Questionnaire (Between 14 and 98: 98 = highest relationship satisfaction).

The effects of massage on mental wellbeing, stress and coping, and relationship satisfaction were compared with mixed ANOVAs. The between groups factor was treatment group (A and B) and the within subjects factor was time-point (T1 and T2). Bonferroni corrected paired-samples T-tests were employed to interrogate significant interaction effects further. See Figure 3 for interaction effects between group and time-point for each test.

#### Mental wellbeing

Mixed ANOVA revealed that there was a significant difference in the WEMWBS score between T1 and T2, *F* (1, 32) = 18.48, *p *< .001, $\eta^{2}$ = .37 but no main effect of group on the WEMWBS score *F* (1, 32) = .58, *p *= .45, $\eta^{2}$ = .02. There was a significant group*time-point interaction effect, *F* (1, 32) = 8.36, *p* = .007, $\eta^{2}$ = .21. Follow up Paired-Samples T-tests revealed that there was a significant difference in group A (PM Treatment) between T1 (mean = 44.2, SD = 6.4) and T2 (mean = 50.4, SD = 7.6), *t* (16) = −4.10, *p *= .001, and that there was no significant difference in group B (Delayed Treatment) between T1 (mean = 44.9, SD = 8.1) and T2 (mean = 46.1, SD = 7.7), *t* (16) = −1.47, *p *= .16.

#### Perceived stress and coping

There was a significant main effect of time-point on the RISCI Stress score, *F* (1, 32) = 6.68, *p *= .015, $\eta^{2}$ = .17, but no main effect of group on the RISCI Stress score *F* (1, 32) = .002, *p *= .96, $\eta^{2}$ = .00. There was a significant group*time-point interaction effect, *F* (1, 32) = 8.38, *p *= .007, $\eta^{2}$ = .21. Paired-Samples T-tests revealed that there was a significant difference for group A between T1 (mean = 17.8, SD = 3.5) and T2 (mean = 14.6, SD = 3.9), *t* (16) = 3.27, *p *= .005, but that there was no significant difference for group B between T1 (mean = 16.2, SD = 3.8) and T2 (mean = 16.4, SD = 4.9), *t* (16) = −.29, *p *= . 78.

There was a significant main effect of time-point on the RISCI Coping score, *F* (1, 32) = 5.31, *p *= .028, $\eta^{2}$ = .14, but no main effect of group, *F* (1, 32) = .999, *p *= .33, $\eta^{2}$ = .03. There was a significant group*time-point interaction effect, *F* (1, 32) = 4.58, *p *= .04, $\eta^{2}$ = .13. Paired-Samples T-tests revealed that there was a significant difference for group A between T1 (mean = 17.3, SD = 3.1) and T2 (mean = 18.9, SD = 3.1), *t* (16) = −3.2, *p *= .006, but that there was no significant difference for group B between T1 (mean = 17.0, SD = 3.6) and T2 (mean = 17.1, SD = 3.3), *t* (16) = − .12, *p *= . 91.

#### Relationship satisfaction

There was no significant main effect of time-point on the PFQ score, *F* (1, 32) = .36, *p *= .55, $\eta^{2}$ = .01. There was no significant main effect of group on the PFQ score *F* (1, 32) = 1.81, *p *= .19, $\eta^{2}$ = .05. The group*time-point interaction approached but did not quite reach statistical significance *F* (1, 32) = 3.80, *p *= .06, $\eta^{2}$ = .11. Interestingly, comparison of the means suggests an increase in the PFQ score in group A compared to a decrease was seen in group B as Figure 3 depicts.

### Analysis 2 sustainability effect

**Figure 4.**
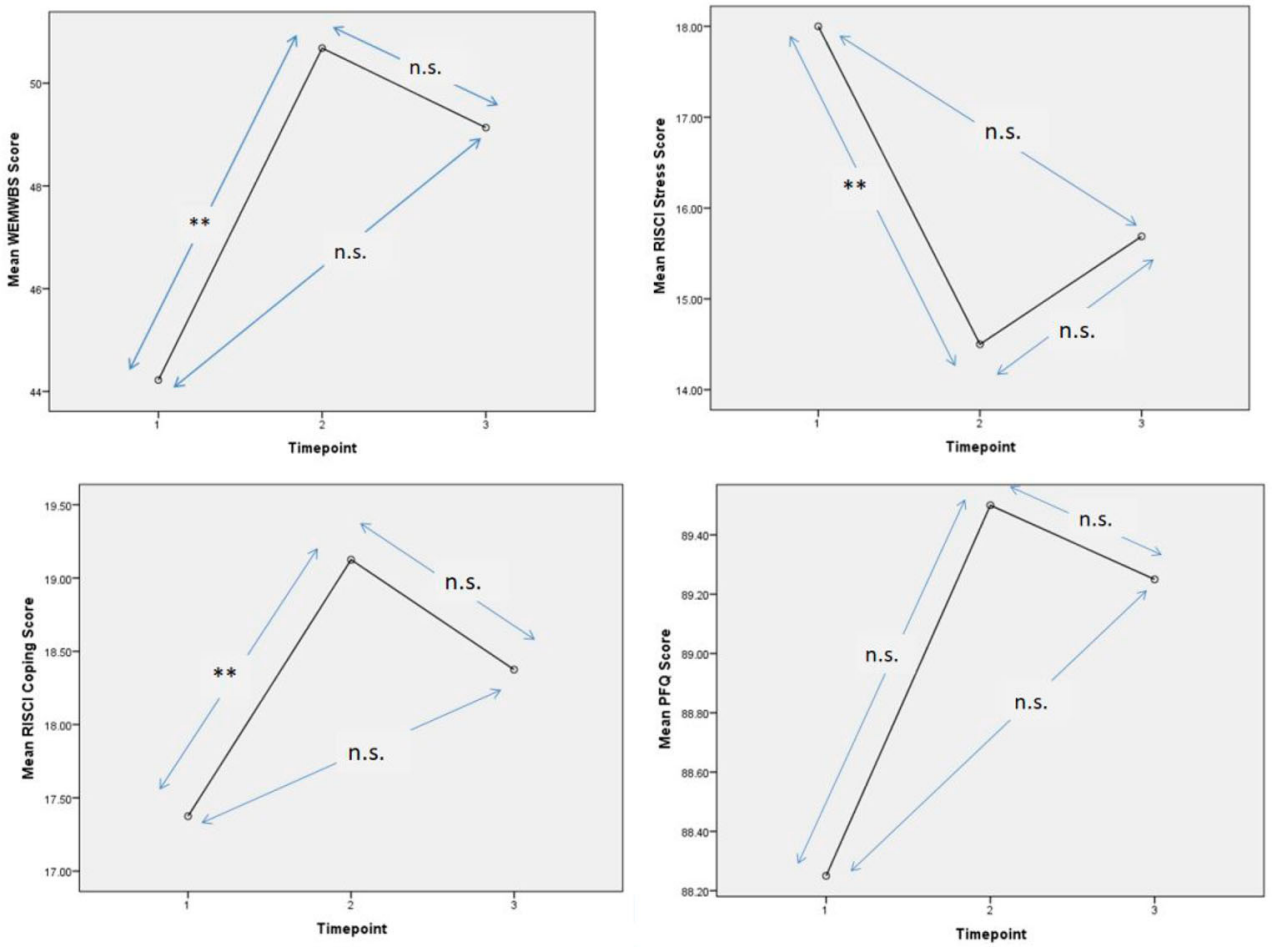
Effect of the PM Programme on the WEMWBS Score, the RISCI Stress and Coping Score, and the PFQ Score at 3 Time-points (T1, T2 &T3) (*N* = 16). **p < .01 n.s.=not significant.

To test the potential lasting effects of the PM programme, data from the WEMWBS, the RISCI, and the PFQ at 3 time-points (including at week 6 follow up) were analysed by repeated measures ANOVA followed by Bonferroni corrected pairwise comparisons for group A only. Group B were not subject to follow up data collection. See Figure 4 for effects of the intervention at 3 time-points.

#### Mental wellbeing

Repeated measures ANOVA revealed that there was a significant effect of time-point on the WEMWBS score, *F* (2, 14) = 8.05, *p = *.005, $\eta^{2}$ = .54. Bonferroni pairwise comparisons revealed a significant increase between ‘before’ (T1, mean = 44.2, SD = 6.6) and ‘after’ the PM programme (T2, mean = 50.7, SD = 7.7) *p = *.003 but interestingly there was no significant difference between T2 and T3 (mean = 49.1, SD = 7.1), *p* = 1., or between T1 and T3, *p = *.10.

#### Perceived stress and coping

There was a significant effect of time-point on the both RISCI Stress and Coping scores, Stress *F* (2, 14) = 6.62, *p = *.009, $\eta^{2}$ = .49, and Coping *F* (2, 14) = 5.63, *p = *.016, $\eta^{2}$ = .45. Bonferroni pairwise comparisons revealed that there was a significant decline in Stress between baseline (T1, mean = 18.0, SD = 3.5) and after massage practice (T2, mean = 14.5, SD = 4.0) *p = *.006, but no significant difference between T1 and T3 (mean = 15.7, SD = 4.8) *p = *.16; or T2 and T3, *p = *.78. Similarly there was a significant improvement in Coping between baseline (T1, mean = 17.4, SD = 3.2) and after massage practice (T2, mean = 19.1, SD = 3.0) *p = *.01, but no significant difference between T1 and T3 (mean = 18.4, SD = 2.6) *p = *.63; or T2 and T3, *p = *1.

#### Relationship satisfaction

There was no significant effect of time-point on the PFQ score, *F* (2, 14) = .49, *p = *.62, $\eta^{2}$ = .07. Bonferroni pairwise comparison revealed that there was a non-significant increase between baseline (T1, mean = 88.3, SD = 9.3) and after massage practice (T2, mean = 89.5, SD = 9.5) *p = *.97, and no significant difference between T1 and T3 (mean = 89.3, SD = 10.5) *p = 1*; or T2 and T3, *p = 1*.

##### Compliance to the massage regime

Table 2 shows the mean frequency of massage practice at home during the three weeks of the programme: group A 5.3 (SD = 2.2), group B 4.8 (SD = 2.4), and total 5.0 (SD = 2.3). This demonstrates that the average number of exchanged massages was 1.7 per week, which met the hypothesised rate in our hypothesis 3.

**Table 2.** Frequency data 1: The weekly mean frequency of massage practice at home during the PM programme (*N* = 38, group A, *n* = 20, group B, *n* = 18).

	Wk1(s.d.)	Wk2(s.d.)	Wk3(s.d.)	Total(s.d.)
Group A	1.9 (.72)	1.8 (.95)	1.6 (.82)	5.3 (2.20)
Group B	2.0 (.84)	1.3 (.97)	1.4 (.98)	4.8 (2.41)
Total	2.0 (.77)	1.6 (.98)	1.5 (.89)	5.0 (2.28)

#### Frequency of practice massage at follow up (T3)

Follow-up data from group A at T3 (week 6) showed, 14 (73.7%) out of 19 participants exchanged massage in both giving and receiving modes after the PM programme finished. The average frequency of massage practice was 5 during post three weeks (i.e. receiving a massage was 4.6 times and giving a massage was 5.4 times during three weeks). The frequency surpassed our hypothesised rate.

### Acceptance

After the PM programme, 25 (73.5%) participants out of 34 expressed their intention to continue exchanging massage at home. Fourteen participants (41%) indicated for 1–2 times a week; 9 (27%) for 1–3 times a month; and 2 (6%) for 3–4 times a week, while 9 (27%) expressed ‘not sure’ and none said ‘no’.

Frequencies showed out of 34, 32 participants (94%) expressed that they would recommend the massage (PM) to their friends and families while 2 (6%) participants were not sure. Responding to the question on how they felt about exchanging massage, 18 participants (53%) indicated definitely positive and 14 (41%) probably positive, while 2 (6%) were not sure and none of the participants indicated negative. Our expectancy in hypothesis 5 was met with high rate of acceptance and willingness to continue PM.

## Discussion

The analyses indicated that the PM programme had a significant positive impact on couples’ mental wellbeing, and perceived stress and coping. This result is consistent with the results previously published relating to emotional stress (Naruse et al., 2018). Furthermore, the current data suggests that these effects might be sustained beyond the length of the programme.

It is notable that such a short educational massage programme (1 h X 3 weeks) appears to provide enough skills and confidence to lay couples such that they can execute massage and deliver significant positive effects on their wellbeing. The results suggest that such skills might be included as contributing to a dyadic coping strategy. A real-world dyadic coping strategy can be empowering for couples and may be particularly important for stressed couples, especially when considering the potential accumulation of chronic daily stress that may lead to infirmities, dysfunctional relationships, and even accelerating ageing (Kiecolt-Glaser, Wilson, & Madison, 2019). Usually couples’ coping strategies in response to life’s stressors develop and operate jointly as a system (Berg & Upchurch, 2007). However, having couple-based coping strategies such as PM that deliver for both parties may be an effective prevention against stress-related illness.

This is not however, thoroughly reflected in the impact on relationship satisfaction, which at first sight might be disappointing. However, the near-significant interaction is associated with a medium to large sized effect (partial eta squared of 0.11, equivalent to Cohen’s d of 0.70). A previous study of fathers massage on their pregnant wives has also revealed a significant effect of massage on dyadic adjustment with a medium sized effect (Cohen’s d = 0.50) (Latifses, Estroff, Field, & Bush, 2005). Looking further into the current data, the failure to reach statistical significance may perhaps be an issue of power, or be due to a ceiling effect since the baseline mean score on this variable was high, leaving little room for increase. It is also possible that the period of the programme may not have been long enough to observe a significant change. It has been shown previously that relationships often require some length of time to develop. Field and colleagues (Field et al., 2008) reported an improvement in relationship quality with a medium sized effect (Cohen’s d = 0.42) for depressed pregnant women as a result of massage by their partners over a period of 16 weeks. Therefore, a longer timescale may be required to demonstrate a positive change in relationship satisfaction through PM. Additionally, we further selected relational factors from the WEMWBS score and re-analysed the total scores of closeness and the perception of being loved. Repeated ANOVA revealed that there was a significant group*time-point interaction effect, *F* (1, 32) = 5.45, *p* = .026, $\eta^{2}$ = .15. Follow up Paired-Samples T-tests revealed that there was a significant difference in group A between T1 (mean = 6.9, SD= .86) and T2 (mean = 7.7, SD = 1.36), *t* (16) = −2.64, *p *= .018, and that there was no significant difference in group B between T1 (mean = 7.1, SD = 1.8) and T2 (mean = 6.9, SD = 1.5), *t* (16) = .46, *p *= .65. Therefore, further investigation of mutual massage effect on relational factors such as perceived closeness, being loved, connectedness and support would be worthy.

Additionally, although not a core aim of the study we went on to consider possible gender effects. Data from group A and B were collapsed and pre and post intervention data analysed with mixed ANOVAs. There were no significant gender differences on the WEMWBS score, *F* (1, 32) = .071, *p *= .79, $\eta^{2}$ = .002. or the RISCI Stress score, *F* (1, 32) = .31, *p *= .58, $\eta^{2}$ = .009, and Coping score, *F* (1, 32) = . 98, *p *= .33, $\eta^{2}$= .030. However, it was revealed that a significant gender*time-point interaction effect was present on the PFQ score, *F* (1, 32) = 4.89, *p *= .034, $\eta^{2}$ = .13.

Paired-Samples T-tests revealed that there was a trend of differences in main effect for female between pre (mean = 80.6, SD = 20.0) and post (mean = 84.4, SD = 15.6), *t* (18) = −1.9, *p *= .07, and that there was no significant difference for male between pre (mean = 86.4, SD = 11.5) and post (mean = 84.2, SD = 11.1), *t* (14) = 1.3, *p *= .23. This gender discrepancy in effects on relationship satisfaction may suggest the usefulness of future studies to explore the most likely population to receive the maximum benefits from such massage i.e. to compare the effect of one-way (e.g. males to female partners) massage and two-way (exchange within partner) massage.

### Sustainability of the PM programme

All the WEMWBS, the RISCI, and the PFQ scores from group A showed no significant differences between T2 (week 3) and T3 (week 6: follow-up survey). These follow-up data indicate the lasting effects of the PM programme on mental wellbeing, perceived stress and coping, and relationship satisfaction 3 weeks after the cessation of the programme. The combined results may suggest that the sustained effect is related to the participants’ continued practice of massage on each other after the cessation of the programme. If this is the case, it may reflect the self-regulatory aspect of PM based on the skills and confidence provided by the programme. An exploration between the compliance rate and the level of mental wellbeing in larger study, as well as a longitudinal study with longer follow-ups would add a deeper understanding regarding this proposal. Since the effects of PM seem to continue after the cessation of the programme, it is possible that the PM programme may provide a cost-effective intervention for mental wellbeing with stress buffering effects. A future cost-effect analysis of the massage programme with input from health economists would add to our understanding.

Bodenmann’s (2005) theory of dyadic coping identifies different forms, including positive supportive dyadic coping (i.e. assisting the other in her/his coping) and common dyadic coping (e.g. relaxing together). Bodenmann explains that positive supportive dyadic coping is not necessarily simply altruistic behaviour, because it involves not only supporting one’s partner but also reduces one’s own stress as well. Mutual massage such as PM can perhaps therefore be regarded as both positive supportive dyadic coping and common dyadic coping.

### Feasibility/acceptance

The simple easily accessible massage (PM) was well accepted by the sample, with 94% of participants indicating that they would recommend PM to their friends and families. This was mirrored by the fact that once the massage course had started, the attrition rate (9.5%) was very low, which is considerably better than for example, a 69% attrition^4^ in a study of caregiver-provided one-way massage for veterans (Kozak et al., 2013). It is encouraging especially when considering the heavy time commitment for participants to attend three classes and complete massage logs nine times over the three weeks without any gift/financial endorsement. After the programme, nearly three quarters (73.5%) of the participants expressed their willingness to continue exchanging massage, while none of the participants indicated negative intentions regarding continuing massage. These positive appraisals are clearly reflected in the data considered earlier. Additionally, and importantly, no physical adversities were reported. However, in answer to the question regarding any disadvantages of exchanging massage, four participants expressed that it was ‘time consuming’ and one explained that ‘after relaxing from just receiving a massage, you get tired again from giving the massage’.

As a consequence of the pleasantness and popularity of the modality (Gulledge et al., 2003), and through the current studies data on intention to continue use, mutual massage can be promoted as a health behaviour among couples along within a concept of ‘selves-care’ (Naruse et al., 2018). The term ‘selves-care’ has been created by the first author referring to activity to care simultaneously for a loved one and oneself. As such, an intervention such as PM might increase perceived coping and reduce perceived stress – an outcome that can only be viewed as positive with regard to wellbeing. Taken with the positive effects on mental wellbeing the PM programme can be an effective dyadic coping intervention with a stress buffering effect.

### Mechanisms of massage

The positive results reported here are argued to be fundamentally due to the power of massage – the expression of caring touch (Pratt & Mason, 1981; Tuohy et al., 2015). The mechanisms behind the impact of massage can be partially explained through biochemical changes that have previously been reported: an increase of oxytocin (Morhenn, Beavin, & Zak, 2012; Riem et al., 2017) and serotonin (Field, Hernandez-Reif, Diego, Schanberg, & Kuhn, 2005), reduction of adrenocorticotropin hormone (Morhenn et al., 2012), and also through increased vagal activity which has been shown to be associated with a decrease in cortisol (Field, 2014; Field et al., 2005). These biochemical changes are potentially deeply entwined with psychological mechanisms: for example, level of oxytocin links with relational wellbeing (Jakubiak & Feeney, 2017), as increased oxytocin influenced positive bonding (Algoe, Kurtz, & Grewen, 2017), empathy (Barraza & Zak, 2009) and trust (Kosfeld, Heinrichs, Zak, Fischbacher, & Fehr, 2005) even during interpersonal conflict (Ditzen et al., 2007).

Empirical studies (e.g. Field et al., 2005; Lindgren et al., 2010; Pinar & Afsar, 2015) also found a reduction of cortisol – the stress hormone – following massage, which may begin to illuminate the links between massage and physical and psychological coping systems. In support of such a proposition, moderate pressure of massage leads to increased blood flow in several brain regions that are involved in stress regulation (Ouchi et al., 2006). These regions include the amygdala and the hypothalamus that regulates autonomic nervous system activity and cortisol secretion. This may explain the stress buffering effect suggested by the results of the current study. Moreover, these brain regions include limbic activity which is linked with emotional regulation (Field, 2014). Emotional regulation – manifest through the impact of positive feelings and relaxation – may enhance not only the receiver’s perception of closeness and support from their partner, but also the giver’s self-efficacy, empathy or understanding of partner’s physical and emotional states. Previous studies of lay massage have found the effects of giving massage on self-efficacy (Kempson & Conley, 2009), self-confidence (Kozak et al., 2013), a significant improvement in relationships (Field et al., 2008) and even in marital adjustment (Latifses et al., 2005).

Such physiological and psychological mechanisms may explain not only the effects of professional therapeutic massage but also those of the kind found here for mutual massage between couples that aims to promote wellbeing in daily life. In order to clarify the mechanisms behind these massage effects, measuring both physiological parameters (e.g. cortisol and oxytocin) and psychological factors (e.g. perceived closeness, support, empathy, and self-efficacy) may be useful.

### Limitations

Time commitment pressures in relation to the online data completion meant that not all participants provided data at all time-points. As a consequence sample sizes for analysis in this study became rather smaller over time. Replication with a larger sample is therefore recommended. A strength of this preliminary study was the study design using an innovative intervention with a delayed treatment component so that all participants were given the opportunity to learn useful massage skills as opposed to employing an inactive control group. However, in contrast, such a study cannot possibly have participants blinded, so demand characteristics are unavoidable. In order to further evaluate the effects of PM and the PM programme rigorously, randomised control trials using an active control group (e.g. relaxation) would be recommended. In addition, the sample in this study may not be representative of general population at large for a number of reasons. Those who responded to the recruitment adverts for the study may be characteristically specific, since they were self-selected volunteers, and as such may be more motivated to be compliant and to deliver positive effects. However, such an intervention would be applicable only to couples who are open and willing to undertake massage, and as such the whole population is not the target at this stage. Qualitative studies investigating how participants experience and perceive PM and the PM programme would be of value by adding a deeper understanding of such a health behaviour and its potential applicability to a wider group. Consideration of motivators and barriers to engagement that such a study could identify can provide important information to improve take up when developing possible health interventions (Moss, Moss, & McInnes, 2018).

## Conclusions

In summary, this experimental preliminary study explored the efficacy of a short educational massage programme in the real-world for couples, and evaluated the subsequent effects of home-based mutual massage. Generally, the hypotheses were supported by these positive results. There were significant improvements in mental wellbeing, perceived stress and coping between group A and B at T2, and there seemed to be sustainable effects of the PM programme on mental wellbeing, perceived stress and coping at week 6. Participants who undertook the PM programme also practiced PM more than once (gave a massage and received a massage) per week at home during the programme and continued exchanging massage thereafter. About three quarters of participants indicated their intentions to continue massage practice, and 94% participants expressed their willingness to recommend of PM to their friends and family. Therefore, the conclusions that can be drawn are that the PM programme delivered significant improvements in mental wellbeing as well as bringing significant reductions in perceived stress and significant improvement in coping among couples. There were sustained effects after the cessation of the massage programme. Healthy but stressed couples could learn and apply massage with satisfaction at home in their daily lives.

In addition to providing new insights, the present findings revealed this alternative aspect of massage function: *massage is not only for therapy, but also for the healthy population to enhance mental wellbeing and provides coping skills and stress buffering effects.* If supporting data can be gained from larger trials mutual self-regulation massage should perhaps be promoted as a positive health behaviour among healthy but stressed couples. This innovative study has laid the groundwork for future studies into the possibility of mutual massage as a preventative dyadic coping strategy for home use to improve overall wellbeing in stressed couples and possibly close relationships in a wider group.

### Suggestions for further study

The current preliminary study posits the effects of lay mutual massage in a position of an early stage feasibility trial; therefore further rigorous studies need to be done to confirm the findings. Suggestions for further studies include: larger randomised controlled trials of mutual massage effects on stress, coping and wellbeing, and relationship satisfaction compared to an active control group; longitudinal study over 12 months exploring the relationships between compliance, wellbeing and relationship satisfaction; explorations of other potential effects of PM on couples relational wellbeing that can be measured by perceived closeness, support, empathy, or self-efficacy; evaluation of the couples massage programme in terms of physiological parameters such as cortisol and oxytocin; qualitative studies to explore how couples experience home-based mutual massage such as PM and the PM programme, in particular regarding motivations and barriers to the use of PM; experimental studies to explore the most likely population to receive the maximum benefits from PM; and cost-effectiveness analysis of the PM programme for potential wider promotion.

## Notes

1 The concept of dyadic coping has been formulated since the early 1990s and a few dyadic coping models have been proposed (e.g. the Systemic-Transaction Model (Bodenmann, 1997)), for details see Falconier, Jackson, Hilpert, and Bodenmann (2015).

2 Generally ‘prevention’ (stop adversities in advance) and ‘intervention’ (treatment) do not coexist in a health modality. However, massage has a unique quality to cover both effects in prevention and in treatment. Preventative intervention in the current article refers to protection from stress related adversities/illness in advance as well as treating perceived stress by coping functions, which is in line with global mental health strategies (World Health Organization, 2013).

3 The measurement of coping is not a measure of coping strategies, but rather a measure of perceived coping effectiveness.

4 The high attrition rate was due to caregivers’ burden. In this study, participants learnt massage by a DVD and were instructed to practice 20 min massage 3 or 4 times a week for 8 weeks.
